# The Predictive Value of Endoglin for Fatal Outcomes in Renal Cell Carcinoma

**DOI:** 10.15586/jkc.v13i4.476

**Published:** 2026-11-06

**Authors:** Munira Hasanbegović, Enisa Ademović, Jelena Kovačević-Prstojević

**Affiliations:** 1 Urology Clinic, Clinical Center University of Sarajevo, Sarajevo, Bosnia and Herzegovina;; 2 Department of Epidemiology and Biostatistics, Faculty of Medicine, University of Sarajevo, Sarajevo, Bosnia and Herzegovina

**Keywords:** endoglin/CD105, fatal outcomes, prediction, renal cell carcinoma

## Abstract

Endoglin (CD105) is an endothelial marker used for detection of tumor, prognostic assessment, and evaluation of the efficacy of chemotherapy protocols. Endoglin is a biomarker involved in angiogenesis, which plays a fundamental role in the growth and metastasis of tumor. To determine the predictive significance of endoglin expression for the occurrence of fatal outcomes in patients with renal cell carcinoma (RCC). Endoglin expression was determined by immunohistochemical staining of endothelial cells in tissue samples obtained from 120 patients who underwent nephrectomy for RCC at the Urology Clinic of the Clinical Center, University of Sarajevo, Bosnia and Herzegovina. The association between CD105 expression and mortality was evaluated over a 5-year follow-up period. In the univariable Cox proportional hazards regression analysis, patients with low and moderate endothelial CD105 expression had an increased risk of death compared to those with high expression, although this association did not reach conventional statistical significance (hazards ratio (HR) = 2.08, 95% Confidence Interval (CI): 1.12–3.85, P = 0.052). In contrast, the multivariable model adjusted for clinicopathological features demonstrated that low and moderate endothelial CD105 expression was independently associated with a significantly higher risk of death, corresponding to a 2.87-fold increase, compared to high expression (HR = 2.87, 95% CI: 1.25–6.59, P = 0.013). Regression diagnostics confirmed the absence of problematic multicollinearity, supporting the robustness of the model. This research demonstrates that endothelial endoglin expression is an independent predictive factor for fatal outcomes in patients with renal cancer. Based on these results, high endothelial endoglin expression is associated with longer survival in patients with RCC.

## Introduction

Endoglin, or cluster of differentiation 105 (CD105), is a crucial endothelial molecular marker (1,2). Located on the cell surface, this transmembrane glycoprotein acts as a co-receptor in the transforming growth factor-beta (TGF-b) receptor complex (3,4). Endoglin serves as a highly specific marker of endothelial activation; notably, endoglin antibodies preferentially target activated endothelial cells engaged in tumoral angiogenesis (5). During the angiogenic process, endoglin plays a critical role in modulating TGF-b signaling pathways (6,7). Functioning as an accessory receptor-modulator, it facilitates essential mechanisms such as cellular migration, endothelial cell proliferation, and capillary sprout formation (8,9).

Comparative studies have demonstrated that endoglin exhibits superior sensitivity and specificity compared to other endothelial markers (10–12). Anti-CD105 antibodies display a significantly higher affinity for proliferating (activated) endothelium, binding to nearly all newly formed endothelial cells and neovessels while reacting with only approximately 20% of the pre-existing non-neoplastic vasculature (13). Consequently, endoglin is an invaluable tool for detection of tumor, prognostic stratification, and the clinical assessment of the efficacy of chemotherapy (14–18).

In spite of the established role of CD105 as a prominent marker of neoangiogenesis, a significant knowledge gap persists regarding its prognostic reliability in renal cell carcinoma (RCC). Although the biological function in endothelial activation is well understood, its precise

impact on long-term survival remains unclear. This uncertainty may partly be explained by findings indicating that the prognostic value of CD105 depends significantly on the cellular compartment in which it is expressed, whether on endothelial or tumor cells.

This study adopts an integrated methodological approach. Unlike previous research focused on univariable associations, we employ a robust multivariable framework to assess the independent predictive significance of endothelial CD105 expression, thereby addressing inconsistencies in the existing literature regarding its prognostic role in RCC. Furthermore, by employing a tertile-based stratification of endothelial CD105 expression, we provide a more granular assessment of its association with 5-year mortality, offering valuable insights for the risk assessment of patient that may complement traditional pathological staging.

## Objective

The aim of the study was to determine the predictive significance of endothelial endoglin expression in relation to mortality in RCC patients.

## Patients and Methods

The study cohort comprised 120 patients, who underwent nephrectomy for RCC at the Urology Clinic of the Clinical Center, University of Sarajevo, Bosnia and Herzegovina, over a period of 2 years. This investigation was conducted as a prospective–retrospective study. Retrospective data collection included medical histories, diagnostic imaging reports, and surgical protocols recorded prior to initiation of study. The prospective evaluation involved the histopathological and immunohistochemical processing of tissue specimens as well as the standardized clinical examination of patients during scheduled follow-up visits and monitoring for tumor recurrence. Only patients with complete medical and histopathological records were included in the final cohort of 120 subjects.

The study was conducted in accordance with the Declaration of Helsinki (19), and ethical approval was granted by the Ethics Committee of the Clinical Center, University of Sarajevo, Sarajevo, Bosnia and Herzegovina (Approval No. 03-02-47492).

The inclusion criteria were as follows:

- Age and gender: Both male and female patients, aged 18–80 years, with an established indication for surgical treatment.
- Surgical eligibility: Absence of absolute contraindications to surgery under general anesthesia.
- Performance status: Eastern Cooperative Oncology Group (ECOG) performance status of 0–3 (20).
- Surgical consent: Written informed consent for surgical procedure.
- Study consent: Written informed consent was obtained from all participants for participation in the study.

Standard histopathological analysis was performed. Tumor tissue samples were fixed in 10% buffered formalin and subsequently embedded in paraffin wax. The paraffin-embedded tissue blocks were sectioned to a thickness of 5 μm using a microtome for hematoxylin and eosin (H&E) staining. The prepared slides were examined microscopically to identify representative tumor tissue sections suitable for the study. Under microscopic guidance, a representative tumor area was marked on the slide, and the corresponding area was identified on the matching paraffin donor block. Cylindrical tumor tissue cores were extracted from each donor block and re-embedded in paraffin to construct 12 multi-tissue blocks. As with the standard paraffin blocks, 4-µm thick sections were cut from multi-tissue blocks. Four slides were prepared from each multi-tissue block: one unstained control slide, one H&E-stained slide, and two sections mounted on Superfrost Plus slides for subsequent immunohistochemical analysis.

Endothelial endoglin expression was quantified via immunohistochemical assessment of immunoreactive endothelial cells. The study used mouse anti-human CD105 monoclonal antibody (clone SN6h, Product Code M3527; Dako Corporation, Glostrup, Denmark) following the manufacturer’s protocol. A brown-colored precipitate at the sites of CD105 antigen expressionwas considered a positive finding, following the completion of immunohistochemical procedure (21).

Microscopic tumor analysis, including assessment of endothelial CD105 expression and determination of microvessel density (MVD), was performed using an Olympus BX40 microscope equipped with a digital camera (resolution of 2448 × 1920 pixels) and the microphotography software. The captured rectangular field measured 660 × 844 μm, corresponding to an area of 557,040 μm^2^ (approximately 0.56 mm^2^). Consequently, vascular elements were counted across two photographs per specimen covering a total surface area of 1.11 mm^2^. This procedure was repeated for three times per slide. Microvessel density was defined as the number of immunohistochemically stained vascular structures within the scanned area of 1.11 mm^2^. In this study, we followed Weidner’s standardized guidelines regarding field selection and counting methodology. Histological sections were initially scanned at low magnification (×10) to identify ‘’hot spots’’ defined as the areas with the highest density of small blood vessels. Subsequently, individual microvessels were counted at higher magnification (×200), and representative images were captured by microphotography. The mean count obtained from three separate fields of view (each representing an area of 1.11 mm^2^) was calculated as the final value for each patient, and this absolute number was used in subsequent statistical analyses. To ensure reproducibility of the immunohistochemical scoring, tissue samples were evaluated independently by two researchers, and the mean vessel count was used for analysis. The researchers were blinded to the clinical and histopathological outcomes of patients. To ensure the reproducibility and accuracy of the scoring process, discrepant or borderline cases were re-evaluated jointly using a multi-head microscope and a shared digital screen. A final consensus score was reached for all cases through mutual discussion prior to statistical analysis.

According to Weidner’s criteria (22), presence of a patent vascular lumen or erythrocytes was not considered mandatory for identification of newly formed microvessels. Thus, the final counts included not only complete patent blood vessels but also endothelial cell cords (strands) and single, isolated endothelial cells.

Following the quantitative counting of endothelial cells by microscopic magnification, tissue samples were categorized into tertiles representing low, moderate, and high endoglin expression, based on the methodologies described by Minhajat et al. (23), Saroufim et al. (24), and Zanjani et al. (25).

Statistical analyses were performed using Microsoft Excel and IBM SPSS Statistics version 27.0 (Statistical Package for Social Sciences [SPSS]). Endoglin expression values were divided into tertiles (low, moderate, and high). The distribution of survival outcomes across these categories was summarized using frequencies and proportions. Associations between clinicopathological variables and survival were initially assessed using Kaplan–Meier analysis and univariable Cox proportional hazards regression. Variables associated with survival at P *<* 0.05 or P *<* 0.10 in univariable analyses, together with clinically relevant covariates, were considered for inclusion in the multivariable Cox proportional hazards model. Multivariable analysis was performed to determine whether endothelial endoglin (CD105) expression retained its prognostic significance after adjustment for potential confounding factors. Hazard ratios (HRs), 95% confidence intervals (95% CIs), and corresponding P values were calculated.

Prior to multivariable modeling, potential multicollinearity among candidate covariates was assessed using Spearman’s rank correlation analysis, χ^2^ testing, tolerance statistics, and variance inflation factors (VIFs). Most predictors demonstrated acceptable VIF values, whereas elevated VIF values were observed for certain pathological tumor (pT) stage categories, reflecting their association with other tumor-related characteristics. Nevertheless, pT stage was retained because of its established prognostic relevance in RCC. Model fit was evaluated using the Omnibus test of model coefficients, the –2 log-likelihood statistic, and Akaike’s Information Criterion (AIC). Statistical significance was defined as P < 0.05. A *post hoc* power analysis was performed to evaluate statistical power of the study.

## Results

The relationship between endothelial endoglin expression and fatal outcomes was evaluated using univariable and multivariable Cox proportional hazards regression over a 5-year follow-up period, from the time of diagnosis until death or censoring. The baseline clinical and pathological characteristics of the study population are summarized in Table 1.

**Table 1:** Clinical and pathological characteristics of the study population (N = 120).

Characteristics	Category	N (%)
Total Patients		**120 (100%)**
Gender	Male	88 (73.3%)
	Female	32 (26.7%)
Age group (years)	30–39	6 (5.0%)
	40–49	12 (10.0%)
	50–59	36 (30.0%)
	60–69	38 (31.7%)
	70–80	28 (23.3%)
ECOG status	0	58 (48.3%)
	1	44 (36.7%)
	2	10 (8.3%)
	3	8 (6.7%)
Tumor size (cm)	<4	48 (40.0%)
	4–6.9	38 (31.7%)
	7–10	23 (19.2%)
	>10	11 (9.2%)
Histological subtype	Clear cell RCC	95 (79.1%)
	Papillary RCC	12 (10.0%)
	Chromophobe RCC	7 (5.8%)
	Other (collecting duct/unclassified)	6 (5.0%)
Pathological tumor stage (pT)	pT1	63 (52.5%)
	pT2	6 (5.0%)
	pT3	46 (38.3%)
	pT4	5 (4.2%)
TNM stage	I	60 (50.0%)
	II	5 (4.2%)
	III	43 (35.8%)
	IV	12 (10.0%)
Fuhrman grade (n = 112)	1	3 (2.5%)
	2	42 (35.0%)
	3	48 (40.0%)
	4	19 (15.8%)
Tumor necrosis	Present	81 (67.5%)
	Absent	39 (32.5%)
Lymphovascular invasion	LVI +	58 (48.3%)
	LVI -	62 (51.7%)
Sarcomatoid/rhabdoid	Present	6 (5.0%)
	Absent	114 (95.0%)
Metastases	Present	28 (23.3%)
	Absent	92 (76.7%)
Endothelial CD105	Low (≤155.6)	40 (33.3%)
expression	Moderate (155.7–305.9)	40 (33.3%)
	High (≥306.0)	40 (33.3%)

Notes. TNM: **T**umor-**N**ode-**M**etastasis (stage); ECOG: Eastern Cooperative Oncology Group.

Tissue samples were stratified into three groups according to the tertiles of the CD105-positive vessel count (Table 2). Endothelial CD105 expression was categorized as low (≤155.6 vessels per 1.11 mm^2^), moderate (155.7–305.9 vessels per 1.11 mm^2^), and high (≥306.0 vessels per 1.11 mm^2^).

**Table 2:** Endothelial expression of endoglin (CD105).

Endothelial CD105 expression	CD105-positive vessel count (endothelial cells) per 1.11 mm^2^	N (%)
Low	≤155.6	40 (33.3%)
Moderate	155.7–305.9	40 (33.3%)
High	≥306	40 (33.3%)
Total		120 (100.0%)

The impact of endothelial CD105 expression levels on patient survival was evaluated using the Kaplan–Meier analysis (Figure 1).

**Figure 1:**
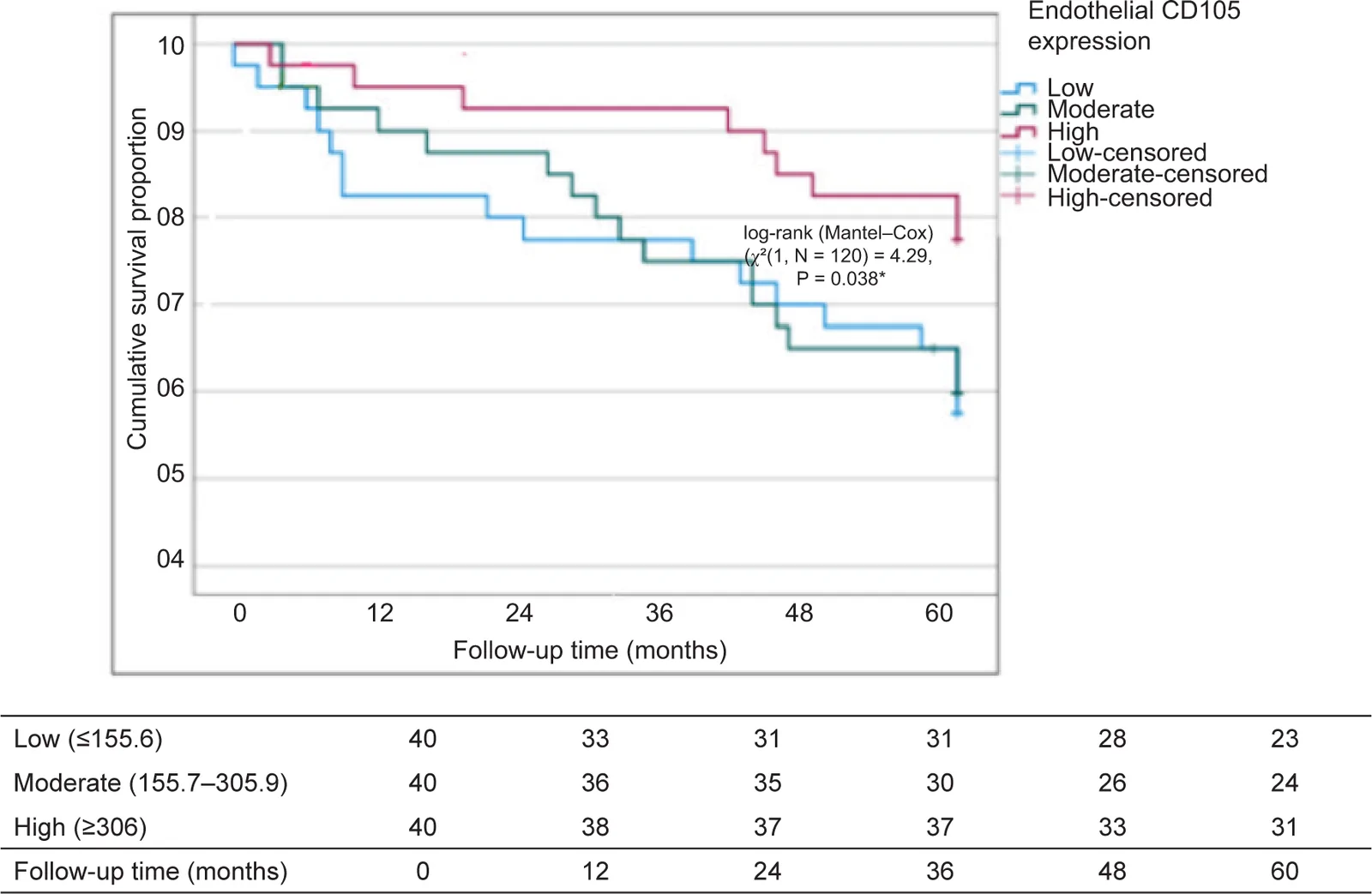
Kaplan–Meier survival curves for the overall survival based on endothelial CD105 expression levels.

Survival was compared between patients with high CD105 expression (≥306 vessels per 1.11 mm^2^) and those with low or moderate expression (<306 vessels per 1.11 mm^2^). The log-rank (Mantel–Cox) test demonstrated a statistically significant difference in survival between the groups (χ^2^(1, N = 120) = 4.29, P = 0.038). Patients with high endothelial CD105 expression (≥306 vessels per 1.11 mm^2^) had a longer mean overall survival (54.75 months) than those with low or moderate endothelial CD105 expression (<306 vessels per 1.11 mm^2^), whose mean survival was 47.58 months. The mean survival period for the overall cohort was 49.97 months (Table 3).

**Table 3:** Mean survival in relation to endothelial CD105 expression.

Endothelial CD105 expression	Overall survival						
	Mean survival (months)	SEM	95% Confidence interval (95% CI)		Log-rank (Mantel–Cox)^†^		
			Lower bound	Upper bound	χ^2^	df	P*
Low and moderate (<306)	47.588	2.239	43.199	51.976	4.292	1	0.038
High (≥306)	54.750	2.268	50.305	59.195			
Total	49.975	1.682	46.677	53.273			

Notes. Endothelial CD105 expression (low and moderate < 306, high ≥ 306); ^†^Log-Rank (Mantel–Cox): test of equality of survival distributions in relation to endothelial CD105 expression; SEM: standard error of mean; ^*^Significance level for two-tailed testing (P < 0.05).

Although the highest survival proportion was observed in the subgroup with high endothelial CD105 expression at the end of 5-year follow-up, no statistically significant difference wasobserved in the overall survival distribution among patients with low, moderate, and high expression levels (P > 0.05) (Table 4).

**Table 4:** Univariable Cox proportional hazards analysis of the overall survival according to endothelial CD105 expression.

Endothelial CD105 expression	Deceased N (%)	Survivors N (%)	Univariable Cox proportional hazards regression analysis		
			HR	95% CI	P value
Low (≤155.6)	17 (42.5)	23 (57.5)	2.12	0.94–4.75	0.069
Moderate (155.7–305.9)	16 (40.0)	24 (60.0)	2.03	0.90–4.60	0.089
High (≥306)	9 (22.5)	31 (77.5)	1.00 (Ref.)	-	-

Notes. CD105 expression values are presented as vessel counts per 1.11 mm^2^.

CI: confidence interval; HR: hazards ratio; Ref.: reference category.

We further investigated differences in mortality risk between patients with high endoglin expression (third tertile) and those with low or moderate expression (first and second tertiles), applying a significance threshold of P < 0.10. During the 5-year follow-up period, nine of the 40 patients (22.5%) in the high endothelial CD105 expression group experienced a fatal outcome, compared to 33 of the 80 patients (41.25%) in the low/moderate expression group.

Utilizing Cox proportional hazards regression with a significance threshold of P < 0.10, patients with low and moderate endothelial endoglin expression exhibited a 2.08-fold higher risk of fatal outcomes because of RCC, compared to those with high expression (HR = 2.08, 90% CI: 1.12–3.85, P = 0.052) (Table 5).

**Table 5:** Univariable Cox proportional hazards regression analysis of the overall survival according to endothelial CD105 expression (high versus low and moderate) at a significance level of P < 0.10.

Endothelial CD105 expression	Deceased N (%)	Survivors N (%)	Univariable Cox proportional hazards regression analysis		
			HR	90% CI	P value
Low and moderate (n = 80)	33 (41.25)	47 (58.75)	2.08	1.12-3.85	0.052
High(n = 40)	9 (22.5)	31 (77.5)	1.00 (Ref.)	–	–

Notes. CI: confidence interval; HR: hazards ratio; Ref.: reference category.

Significance level was set at *P* < 0.10 for two-tailed testing.

To identify independent prognostic determinants, a multivariable Cox proportional hazards regression was performed, incorporating clinicopathological variables (pT stage, tumor size, tumor necrosis, lymphovascular invasion, rhabdoid and sarcomatoid components, and metastases) together with CD105 expression. Variables demonstrating a significant or borderline association with survival in univariable analysis at thresholds of P < 0.05 and P < 0.10 were entered into the model.

Collinearity diagnostics were performed using VIF and tolerance values according to established guidelines [26]. VIF values > 10 were indicative of severe multicollinearity, whereas VIF values of 5–10 were considered indicative ofmoderate collinearity. Most predictors demonstrated acceptable VIF values (1.08–4.10), indicating no substantial concerns regarding collinearity. In contrast, pT stage categories (except pT4) showed elevated VIF values, ranging from 7.6 to 17.0, consistent with substantial collinearity with other pathological features. Despite this, pT stage was retained in the final Cox regression model because of its established clinical importance. Endothelial CD105 expression demonstrated a VIF of 1.10, confirming the absence of collinearity.

Within this adjusted multivariable framework, endothelial CD105 expression was independently associated with the overall survival. Patients with low and moderate expressions had a 2.87-fold higher risk of death than those with high expression, which served as the reference category (HR = 2.87, 95% CI: 1.25–6.59, P = 0.013) (Table 6).

**Table 6:** Multivariable Cox proportional hazards regression analysis of the overall survival according to endothelial CD105 expression.

Endothelial CD105 expression	Multivariable Cox proportional hazards regression analysis		
	HR	95% CI	P value
High	1.00 (Ref.)	–	–
Low and Moderate	2.87	1.25–6.59	0.013

Notes. Adjustments were made for covariates, including pathological tumor (pT) stage, tumor size, tumor necrosis, lymphovascular invasion, rhabdoid/sarcomatoid differentiation, and distant metastases. CI: confidence interval; HR: hazards ratio; Ref.: reference category.

Statistical significance was defined as P < 0.05.

Model performance: Omnibus test, P < 0.001; Akaike information criterion (AIC) = 369.22; CD105 variance inflation factor (VIF) = 1.10.

The global multivariable model demonstrated high statistical significance, as indicated by the Omnibus test (P < 0.001). Within this model, endothelial CD105 expression emerged as a significant independent predictor of the overall survival (HR = 2.87, 95% CI: 1.25–6.59, P = 0.013). The final model had a –2 log-likelihood value of 341.216 and an Akaike information criterion (AIC) of 369.22. A *post hoc* power analysis was performed for Cox proportional hazards regression model. The analysis included 120 patients, 42 observed events, and 8 covariates. The primary predictor demonstrated an HR of 2.87 (P = 0.013), and the final model showed a -2 log-likelihood value of 341.216. Based on the observed effect size, number of events, sample size, and an alpha level of 0.05, the estimated statistical power was approximately 80%. The limited events-per-covariate ratio (5.25) should be considered when interpreting the model because of the potential risk of overfitting.

## Discussion

Endoglin offers several advantages over pan-endothelial markers (27, 28). Various studies have shown that it is far more sensitive and specific than other endothelial markers used to date (11, 12). Currently, there has been a shift from individual clinical prognostic factors toward advanced integrated multivariable models that incorporate molecular and genetic information (29). The use of molecular markers promises significantly greater precision in the prognostic assessment of RCC (30, 31).

This study highlights endothelial CD105 expression as an independent prognostic marker of fatal outcomes in RCC patients. The methodological approach—using Cox proportional hazards regression with adjustment for all clinicopathological variables significant in univariable analysis—ensured that the observed association was not confounded by established predictors, such as pT stage, tumor necrosis, or metastatic status. By including endothelial CD105 expression, despite its borderline univariable significance, the analysis reflects the biological plausibility of this marker and demonstrates its independent contribution when evaluated in a multivariable context. Importantly, stratification by expression levels revealed distinct survival outcomes, with patients exhibiting high endothelial CD105 expression differing from those with low and moderate expression, underscoring the clinical relevance of biomarker intensity (32, 33).

The current literature presents a notable paradox regarding CD105 in RCC. On the one hand, several studies have linked high CD105-assessed microvessel density with aggressive tumor behavior and poor survival rates, identifying it as a marker of high metastatic risk. Conversely, other researchers have reported that higher endothelial expression of CD105 may paradoxically correlate with a more favorable prognosis, potentially because of its role in vascular maturation or tissue stabilization. Such discrepancies between these studies on endoglin expression and survival may reflect differences in tumor biology, patient cohorts, or methodological approaches, highlighting the need for standardized assessment of endothelial markers (34). A critical factor in resolving the conflicting findings in the existing literature is the precise distinction between the cellular compartments in which CD105 is expressed.

While the results of studies investigating the prognostic implications of endothelial CD105 expression remain divergent, evidence has shown that tumor-cell CD105 expression carries negative prognostic significance. Specifically, high CD105 expression within the tumoral compartment strongly correlates with advanced tumor stage, higher nuclear grade, and poor overall survival (35). In our study, we focused exclusively on endothelial CD105 expression, thereby avoiding the confounding effects of global CD105 upregulation.

Endothelial CD105 expression carries several key clinical implications for RCC. First, incorporating CD105 into multivariable models improves risk stratification beyond traditional clinicopathological factors, helping to identify high-risk patients who require aggressive follow-up or adjuvant therapy. Second, owing to its superior sensitivity over pan-endothelial markers such as CD31 or CD34, endoglin provides highly specific detection of active tumor angiogenesis. Furthermore, as a robust marker of proliferating endothelium, CD105 serves as a promising target for selective anti-angiogenic therapy, aiding in overcoming therapeutic resistance by blocking alternative survival pathways within the tumor microenvironment (36–38). Finally, it represents a valuable tool for therapeutic assessment, acting as a dynamic biomarker to monitor clinical response to anti-angiogenic treatments.

A significant correlation between the endothelial expression of endoglin and patient survival is cited in a study conducted by Saroufim et al. (24). The study highlights that the prognostic value of CD105 depends on identifying whether expression occurs within the vascular or tumoral compartment. The cited study analyzed the data of 102 patients with clear-cell RCC. The study showed that patients with high endothelial CD105 expression had an apparently better 5-year survival rate (91% versus 58%, P = 0.001). This parameter was an independent prognostic factor. Instead, tumoral CD105 expression on cancer cells strongly correlated with advanced tumor stage and nuclear grade, serving as an independent prognostic marker for poor overall survival and unfavorable prognosis (P = 0.002). Tumoral CD105 expression increased the risk of death by more than three times.

The results of this study regarding improved survival in the high endothelial expression group were consistent with the findings of Sandlund et al. (39). In their study of 210 patients, which included 168 individuals with clear cell RCC, the authors reported that high endothelial endoglin expression correlated with a more favorable prognosis (P *=* 0.04). Specifically, Sandlund et al. observed that endoglin expression was inversely correlated with tumor stage (P = 0.008) and nuclear grade (P = 0.01) in this specific cohort, explaining the association between higher expression and favorable clinical outcome (39).

Conversely, Ollauri-Ibáñez et al. demonstrated that continuous endothelial endoglin overexpression paradoxically disrupts angiogenesis and inhibits tumor growth by preventing vascular maturation (40). Simultaneously, however, continuous endoglin overexpression facilitates metastases, thereby shortening patient survival. This finding highlights a nuance that challenges the traditional view of endoglin solely as a pro-angiogenic marker highly upregulated in proliferating tumor vasculature (40).

In contrast to our findings, Dubinski et al. reported that higher endothelial CD105 expression correlates with advanced tumor stages and decreased survival in patients with RCC (41). Their work underscores how advancements in the digital assessment of immunohistochemical markers can help standardize the evaluation of tumor biomarkers (41).

Similarly, a study conducted by Zanjani et al., which included 186 patients, demonstrated that endothelial and tumoral cytoplasmic endoglin serve as a significant, subtype-specific prognostic biomarker for clear cell RCC, where higher expression signals aggressive tumors and lower survival rates (25). Conversely, no significant association was found between CD105 expression and clinicopathological parameters or prognosis in papillary and chromophobe RCC. Although CD105 expression correlates with poorer outcomes, it was not an independent predictor of survival, potentially reflecting the influence of established risk factors in multivariable analysis (25).

The robustness of our model confirmed by low collinearity supports the validity of these findings. While other variables, such as pT4 stage and tumor necrosis, also retained prognostic relevance, their detailed analysis will be presented separately to maintain focus on endothelial endoglin expression. Overall, this methodological strategy underscores the imcaportance of integrating molecular biomarkers into survival models, complementing traditional pathological features and potentially refining patient risk stratification (42, 43).

The current study has certain limitations. First, the relatively low number of outcomes relative to the number of covariates in multivariable model introduces a risk of overfitting. Therefore, the estimated HRs should be interpreted with caution. Second, endothelial CD105 expression demonstrated only borderline statistical significance in univariable analysis. Nevertheless, its association with patient outcomes remained statistically significant after adjusting for established clinicopathological prognostic factors, suggesting that endothelial CD105 expression may be an independent prognostic factor. Further studies in larger prospective cohorts are required to confirm these observations. Finally, the hybrid prospective–retrospective observational study relies partly on the existing medical records, which have inherent risk of selection and reporting bias compared to a purely prospective trial.

## Conclusion

Endothelial endoglin expression is an independent prognostic factor in RCC when evaluated within a multivariable Cox regression framework adjusted for established clinicopathological predictors. Patient stratification by expression intensity provides distinct prognostic value beyond conventional pathological variables. Although previous literature reports conflicting associations between endothelial endoglin expression and clinical outcome, our findings reinforce its potential as a reliable biomarker. Integrating endothelial endoglin expression into prognostic models may refine risk stratification and guide future translational research. Ultimately, validation in larger, independent prospective cohorts and dedicated mechanistic studies are required to confirm these observations and to translate them into clinical practice.
